# History of Suicide Attempt Is Associated with Reduced Medial Prefrontal Cortex Activity during Emotional Decision-Making among Men with Schizophrenia: An Exploratory fMRI Study

**DOI:** 10.1155/2018/9898654

**Published:** 2018-03-01

**Authors:** Stéphane Potvin, Andràs Tikàsz, Stéphane Richard-Devantoy, Ovidiu Lungu, Alexandre Dumais

**Affiliations:** ^1^Centre de Recherche de l'Institut Universitaire en Santé Mentale de Montréal, Department of Psychiatry, University of Montreal, 7331 Hochelaga, Montreal, QC, Canada H1N 3V2; ^2^Department of Psychiatry & Douglas Mental Health University Institute, McGill Group for Suicide Studies, McGill University, Montreal, QC, Canada; ^3^Centre de Recherche de l'Institut Universitaire de Gériatrie de Montréal, 4565 Chemin Queen-Mary, Montreal, QC, Canada H3W 1W5; ^4^Centre for Research in Aging, Donald Berman Maimonides Geriatric Centre, 5795 Caldwell Avenue, Montreal, QC, Canada H4W 1W3; ^5^Institut Philippe-Pinel de Montréal, 10905 Henri-Bourassa, Montreal, QC, Canada H1C 1H1

## Abstract

Despite the high prevalence of suicidal ideas/attempts in schizophrenia, only a handful of neuroimaging studies have examined the neurobiological differences associated with suicide risk in this population. The main objective of the current exploratory study is to examine the neurofunctional correlates associated with a history of suicide attempt in schizophrenia, using a risky decision-making task, in order to show alterations in brain reward regions in this population. Thirty-two male outpatients with schizophrenia were recruited: 13 patients with (SCZ + S) and 19 without a history of suicidal attempt (SCZ − S). Twenty-one healthy men with no history of mental disorders or suicidal attempt/idea were also recruited. Participants were scanned using fMRI while performing the* Balloon Analogue Risk Task*. A rapid event-related fMRI paradigm was used, separating decision and outcome events, and the explosion probabilities were included as parametric modulators. The most important finding of this study is that SCZ + S patients had reduced activations of the medial prefrontal cortex during the success outcome event (with parametric modulation), relative to both SCZ − S patients and controls, as illustrated by a spatial conjunction analysis. These exploratory results suggest that a history of suicidal attempt in schizophrenia is associated with blunted brain reward activity during emotional decision-making.

## 1. Introduction

Compared to the general population, the life expectancy of patients with schizophrenia is approximately 20 years shorter [1]. Unfortunately, suicidal behavior is one of the key factors contributing to this dramatically reduced life expectancy [2]. Indeed, large-scale empirical evidence has shown that between 15 and 35% of schizophrenia patients will attempt to commit suicide (mostly during the first years following the onset of the disorder) and that 5-6% will end their life by suicide [3, 4].

During the last decades, several clinical studies have examined the risk factors for suicidal behavior in schizophrenia. Overall, studies have shown that most risk factors for suicidal behavior in schizophrenia are largely similar to those identified in nonpsychotic individuals with a history of suicidal behavior. Large cohort studies as well as large-scaled systematic reviews (>70 studies) have shown that suicidal behavior in schizophrenia is consistently linked with mood instability, hopelessness, suicidal ideation, previous suicidal attempt, self-injurious behavior, impulsivity, and substance use disorders [3, 5, 6]. Some studies have shown that hallucinations and better insight into the disorder are risk factors for suicidal behavior in schizophrenia, but these findings have not been replicated consistently [5, 7].

In nonpsychotic individuals, cognitive studies have highlighted strong relationships between suicidal behavior and poor emotional decision-making [8]. The emotional decision-making deficits of individuals who engage in suicidal behaviors are hypothesized to result from dysfunctions of the brain reward system. Emotional decision-making relies on mental processes that are mostly driven by reward seeking, as it involves the selection of a behavior based on predicted positive or negative outcomes [9]. Findings from a large-scale meta-analysis of 206 functional imaging studies performed among healthy individuals indicate that rewards activate a valuation system composed of the (ventro-) medial prefrontal cortex (mPFC), the (ventral) anterior cingulate cortex, and the (ventral) striatum [10], all key regions of the brain reward system. This is consistent with recent fMRI studies highlighting the crucial role of these reward-related regions in emotional decision-making [11]. In nonpsychotic individuals, mounting evidence from structural and functional imaging studies suggest that suicidal behaviors are associated with alterations in these reward-related brain regions [12–14].

Despite the high prevalence of suicidal ideas/attempts in schizophrenia, only a handful of neuroimaging studies have examined the neurobiological differences associate with suicide risk in this population. Thus far, most of these studies used structural neuroimaging, and the very few functional neuroimaging studies available have administered tasks measuring cognitive control, not risky decision-making [15–17]. The main objective of the current study is to examine the neurofunctional correlates associated with a history of suicide attempt in schizophrenia, using a risky decision-making task, in order to show alterations in brain reward regions in this population.

## 2. Methods

### 2.1. Participants

Thirty-two male outpatients with schizophrenia (DSM-IV criteria; age 18–57 years) were recruited: 13 patients with (SCZ + S) and 19 without a history of suicidal attempt (SCZ − S). Diagnoses were established with the* Structured Clinical Interview for DSM-IV* [18]. Twenty-one healthy men with no history of mental disorders or suicidal attempt/idea were also recruited. History of suicidal attempts/ideas was evaluated with the* Columbia Suicide Severity Rating Scale* [19] and review of medical records. Participants had no concomitant neurological disorders, substance use disorders (last 12 months), or magnetic resonance imaging (MRI) contraindications and had an estimated IQ over 70, as evaluated by the* Wechsler Abbreviated Scale of Intelligence* [20]. Participants tested negative at a urine drug screening administered before the fMRI session. Symptoms severity was evaluated with the* Positive and Negative Syndrome Scale* (PANSS) [21], and impulsivity was evaluated with the* Barratt Impulsiveness Scale* (BIS) [22]. Schizophrenia participants were treated with one or more of first- and/or second-generation antipsychotics (see Table 1). The influence of antipsychotics was examined by calculating olanzapine equivalents [23]. All participants signed a detailed consent form. The study was approved by the ethics committee from the* Institut Philippe-Pinel de Montreal* and the* Unité de Neuroimagerie Fonctionnelle*.

### 2.2. Functional Task

The* Balloon Analog Risk Task* (BART) was administered in the scanner. The BART measures risky decision-making by presenting participants with a balloon that can be incrementally inflated by pressing a button. Each inflation adds virtual money into the participant's account up to a threshold at which point the balloon explodes. With each button click, participants must choose whether to cash out or to take the risk of inflating the balloon and potentially earn more money. The event-related functional MRI (fMRI) adaptation of the BART allows the examination of decision and outcome periods separately and of associations between brain responses and objective risks, based on predetermined probabilities of explosions [24]. The BART was chosen, since it is simple and easy to understand and relatively independent of learning effects, which are likely to have influenced schizophrenia patients' performance on alternative decision-making tasks which typically require repeated calculations [25].

The task was administered in two 8 min runs, which began and ended with display of a fixation cross at the center of the screen to establish baseline activity. At the beginning of each trial, the screen displayed an image of a balloon. For a given balloon trial, 12 successive inflations were possible. The probability of explosion over successive inflations increased parametrically. For each balloon, participants had no time restrictions to make a decision (i.e., to choose to Inflate the balloon or Cash In the wager). Each response was followed by an interstimuli interval, jittered and randomized, and lasting 0 to 6 seconds. During this delay, no feedback was given to participants. Following an inflation of the balloon, the outcome (Success or Explosion) was presented (for 2000 ms). If the balloon was inflated, participants were allowed to make the next decision after a delay of 1500, 2000, or 2500 ms. After a decision to Cash In, the balloon was replaced by the words “You Win!” (for 2000 ms). After a Win or an Explosion, the screen became black (for 2000, 3000, or 4000 ms), and a new trial was presented. Total earnings were calculated.

### 2.3. Neuroimaging Acquisition Parameters

Whole-brain fMRI was performed using an echo-planar imaging sequence measuring Blood Oxygenation Level Dependent (BOLD) signal (TR = 2090 ms; TE = 30 ms; FA = 90°; FoV = 224 mm; matrix = 64 × 64; voxel size = 3.5 mm^3^; 38 axial slices) on a Siemens TIM TRIO MRI system, using a 32-channel, high-resolution, transmit/receive brain volume coil. An inline retrospective motion correction algorithm was employed while the EPI images were acquired. Individual high-resolution coplanar anatomical images were also acquired during the same scanning session (three-dimensional, ultrafast gradient echo sequence; TR = 2300 ms, TE = 2.98 ms; FA = 90°; FoV = 256 mm; matrix size = 256 × 256; voxel size = 1 mm^3^; 176 sagittal slices).

### 2.4. Neuroimaging Analyses

fMRI data was analyzed with BrainVoyager QX 2.8 (Brain Innovation, Maastricht, Netherlands) software. Functional images were corrected for differences in slice-time acquisition and for motion artifacts (≤2 mm), high-pass filtered, coregistered to the corresponding anatomical image, then spatially normalized to the Talairach space [26], and spatially smoothed with a 3D isotropic Gaussian kernel (8 mm FWHM).

An event-related approach was employed for data analysis. The BART model included five experimental events, namely, the decision to Inflate or to Cash In, and the Success, Win, and Explosion outcomes. The predictors were entered as fixed factors in a single-subject general linear model (GLM), and an autoregressive AR(2) model was used to account for serial correlations. The AR(2) model has been shown to outperform AR(1) and is the current standard in BrainVoyager QX 2.8 [27, 28]. Explosion probabilities (*z*-transformed) were included as parametric modulators for the Inflate and Success events [24, 29]. Then, a second-level analysis corresponding to a random-effect GLM was used for group comparisons [30]. The focus of the group level analyses was the parametrically modulated events (i.e.,* Inflate* and* Success*) [24, 29]. Spatial conjunction analyses were performed to identify neurofunctional alterations common to the [SCZ + S versus SCZ − S] and [SCZ + S versus healthy men] group comparisons.

The statistical threshold for significance was determined by Monte Carlo simulation [31]. Assuming a voxel-level threshold of *p* < 0.001 (10,000 simulations), a minimum cluster size of 343 mm^3^ was required to correct for multiple comparisons at *p* < 0.05, brain-wise. We also performed correlation analyses between regional BOLD responses and clinical variables. The MATLAB NeuroElf toolbox (http://neuroelf.net) and the ggplot2 plotting system for R (http://ggplot2.org) were used for visualization. The open-source image editor GIMP (http://www.gimp.org) was used to build the figure.

## 3. Results

### 3.1. Clinical Variables

Men with schizophrenia (SCZ + S and SCZ − S) had lower IQ and higher levels of impulsivity (BIS total score) than healthy men. We observed no differences in handedness and ethnicity and total earning on the BART between the three groups. SCZ + S patients were older than SCZ − S patients and healthy men. The two groups with schizophrenia did not differ in terms of psychiatric symptoms (PANSS) and impulsivity (BIS). There was a trend towards significance in the case of olanzapine equivalents (see Table 1).

### 3.2. fMRI Data

#### 3.2.1. Activations in Healthy Men

During the* Inflation* event, a one-sample *t*-test revealed that the explosion risk level was positively associated with activity in the bilateral insula, while being negatively associated with activity in the left precentral gyrus and parietal, temporal, occipital, and cerebellar regions (see Table 2). During the Success event, significant activations were observed in healthy men in several frontal, cingulate, limbic, striatal, parietal, occipital, and cerebellar regions, including the medial prefrontal cortex, the bilateral insula, the anterior cingulate cortex, and the (right) putamen (see Table 2).

#### 3.2.2. Between-Group Differences

For the* Inflation* event, between-group comparisons revealed that SCZ + S patients had increased activations in the right cerebellar declive and lingual gyrus in comparison to SCZ − S patients, as well as increased activations in the left superior temporal gyrus in comparison to healthy participants. There were no differences between SCZ − S patients and healthy participants (see Table 3). Spatial conjunction analyses for this event did not reveal a pattern of BOLD signal specific to SCZ + S patients; that is, we observed no neurofunctional alterations* common* to both SCZ + S versus SCZ and SCZ + S versus healthy men group comparisons [note that across the 3 groups, significant activations were observed during the Inflation event in the right (*x* = 39; *y* = 17; *z* = 4; *t* = 6.7; *p* < 0.001; 3700 mm^3^) and left insula (*x* = −33; *y* = 20; *z* = 7; *t* = 5.2; *p* < 0.001; 909 mm^3^) and the right superior frontal gyrus (Brodmann area 8: *x* = 6; *y* = 29; *z* = 46; *t* = 4.1; *p* < 0.001; 423 mm^3^)].

**Figure 1:**
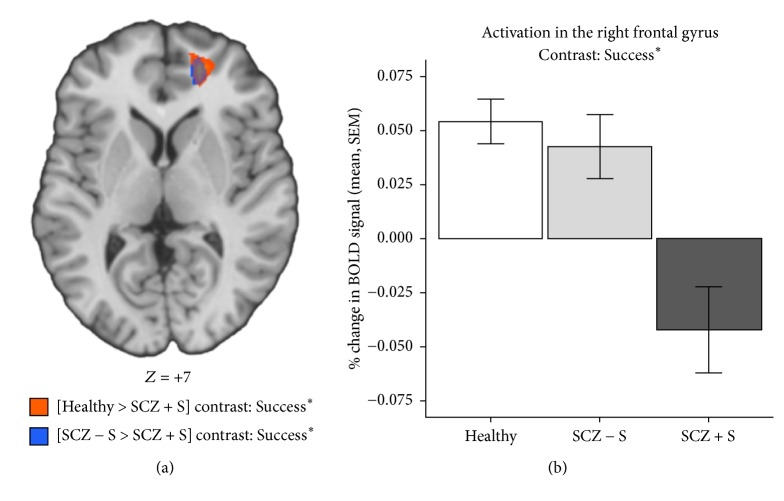
Group differences in BOLD responses of the right frontal gyrus during the success event. BOLD = blood oxygen level dependent; SCZ + S = schizophrenia with lifetime suicidal attempt; SCZ − S = schizophrenia without violent behavior or suicidal attempt; SEM = standard error of the mean; ^*∗*^parametrically modulated.

For the* Success* event, SCZ + S patients also had decreased activations in the right medial frontal/anterior cingulate gyrus, cuneus, and cerebellar culmen and left middle occipital gyrus, in comparison to healthy men. Likewise, SCZ + S patients had decreased activations in the right medial frontal/anterior cingulate gyrus, relative to SCZ − S patients (see Table 4). Spatial conjunction analyses revealed that SCZ + S patients had significantly (*p* < 0.001) decreased activations specifically in the right medial frontal gyrus in comparison to* both* SCZ − S and healthy participants (Figures 1(a) and 1(b)). Covariance analyses indicated that age and medication did not influence this result. Furthermore, the individual parameter estimates of the medial frontal gyrus did not correlate with psychiatric symptoms, antipsychotic medication, or impulsivity among men with schizophrenia.

## 4. Discussion

To our knowledge, this is the first fMRI study seeking to determine if brain reward alterations are associated with a history of suicidal attempt in schizophrenia, using a risky decision-making task. In healthy men, activations were observed in the medial prefrontal cortex, the anterior cingulate cortex, and the right putamen during the* Success* event and in the bilateral anterior insula during both* Inflation* and* Success* events, as shown in previous fMRI studies using the BART [24, 29, 32, 33]. The most important finding of this study is that SCZ + S patients had reduced activations of the mPFC during the Success event (with parametric modulation), relative to both SCZ − S patients and controls, as illustrated by the spatial conjunction analyses. In the past, two fMRI studies performed in nonpsychotic individuals using the BART have shown that the mPFC activations increase parametrically with risk level during the Success event [32, 33]. Given the fact that mPFC is a key region of the brain reward circuitry [10], the parametric increase in mPFC activity has been proposed to reflect a risk-related increase in the rewarding value of the Success outcome. Bearing this in mind, the main result of the current study shows that the rewarding value of Success outcomes does not increase when risk increases in SCZ + S patients, which confirms our a priori hypothesis that a history of suicide behavior in schizophrenia is associated with brain reward (e.g., mPFC) alterations. More precisely, we found a* blunted* brain reward response in SCZ + S patients, not a sensitized one. As such, our main result is consistent with the facts that blunted brain reward activity results in anhedonia (e.g., reduced ability to experience pleasure) [34] and that anhedonia is associated with suicidal behavior in schizophrenia, as shown in a 14-year longitudinal study involving 150 patients [35].

Other differences in brain activations were observed between groups, notably during the decision to Inflate the balloon. During this event, SCZ + S patients had increased activations in the right cerebellar declive and the lingual gyrus, relative to SCZ − S patients, as well as increased activations on the left superior temporal gyrus, relative to healthy controls. These results are difficult to interpret, however, given that the cerebellum, the lingual gyrus, and the superior temporal gyrus are not known to play a key role in reward processing [10] and emotional decision-making [11]. Moreover, none of the differences were specific to the SCZ + S group, as the spatial conjunction analyses revealed no neurofunctional alterations that were common to the comparisons of SCZ + S patients to* both* SCZ − S patients and healthy controls. Moreover, all three groups activated the bilateral (anterior) insula and the superior frontal gyrus, which have been shown, in past fMRI studies using the BART, to be activated during the Inflation event [29, 36]. Taken together, the results of the current study suggest that suicidal behavior in schizophrenia is more closely associated with neurofunctional alterations during reward receipt than during decision-making per se.

Despite its novelty, the current study has several limitations. The most important limitation is that the group of SCZ + S patients was relatively small. Also, study participants comprised only males, thus limiting the generalization of results to one of the sexes. However, the influence of sex on brain reward processing in schizophrenia is relatively unknown [37]. In addition, our results may have been influenced by age differences, that is, by the fact that SCZ + S patients were older than participants in the other 2 groups. Although the changes occurring in the brain of schizophrenia patients when they grow older are insufficiently studied, some (mixed) evidence suggests that the frontal lobe is sensitive to the aging process in schizophrenia [38]. However, we included age as a covariate in the analyses and found that the difference in mPFC activity remained significant. Finally, it is also possible that our results have been influenced by the effects of antipsychotic treatment. Indeed, there was a trend towards higher antipsychotic dosage in the SCZ + S group compared to the SCZ − S group. Given that antipsychotics share the common property of blocking dopamine-D_2_ receptors [39] and that dopamine obviously plays a key role in rewarding processing [40], it is only reasonable to postulate that our results may be partially explained by the effects of antipsychotics. However, we performed analyses using olanzapine equivalents as covariates and found that antipsychotic dose had no influence on results. Importantly, one needs to consider that clinicians may have increased antipsychotic dosage in SCZ + S patients simply in order to prevent additional attempts. The limitations of study are out-weighted by the need to study this issue in schizophrenia and by the fact that these patients are at high risk of ending their life by suicide, while the neuroscientific research has fundamentally ignored this crucial clinical issue.

## 5. Conclusion

In this exploratory fMRI study, we found that a history of suicidal attempt in schizophrenia is associated with blunted brain reward activity during emotional decision-making. Future studies will need to replicate the current main finding in a larger group of SCZ + S patients, while focusing on the processing of reward receipt and paying attention to sex and age differences. Attention will also need to be paid to antipsychotic dosage.

## Figures and Tables

**Table 1:** Participant characteristics.

	SCZ + S (*n* = 13)	SCZ − S (*n* = 19)	Healthy controls (*n* = 21)	Significance
Age, mean (SE)	39.1 (2.2)	32.1 (2.1)	32.1 (1.8)	*F* = 3.14; *p* = 0.05^*∗*^
Handedness, % right	72.7	100.0	95.0	*χ* ^2^ = 7.91; *p* = 0.10
Ethnicity, % Caucasian	92.3	84.2	90.5	*χ* ^2^ = 5.19; *p* = 0.27
IQ, (SE)	93.6 (4.6)	89.2 (2.1)	101.2 (3.5)	*F* = 3.48; *p* = 0.04^*∗∗*^
BART total $ (SE)	17.9 (1.2)	18.1 (1.2)	19.4 (1.1)	*F* = 0.48; *p* < 0.62
BIS total (SE)	67.0 (3.0)	63.7 (2.2)	52.9 (1.7)	*F* = 11.72; *p* < 0.001^*∗∗∗*^
Diagnoses	5 SA	2 SA	-	*χ* ^2^ = 3.23; *p* = 0.07
Age of onset (SE)	26.7 (3.5)	21.1 (1.1)	-	*t* = −1.53; *p* = 0.15
PANSS				
Positive	17.0 (1.0)	16.1 (0.5)	-	*t* = −0.92; *p* = 0.36
Negative	16.2 (0.8)	17.5 (1.4)	-	*t* = 0.77; *p* = 0.44
General	37.1 (1.8)	35.7 (1.4)	-	*t* = −0.59; *p* = 0.56
Clozapine, %	46.2	55.6	-	*χ* ^2^ = 0.27; *p* = 0.61
Olanzapine equivalents mg (SE)	18.9 (3.3)	11.5 (1.4)	-	*t* = −2.07; *p* = 0.06

BART = Balloon Analogue Risk Task; BIS = Barratt Impulsiveness Scale; PANSS = Positive And Negative Syndrome Scale; IQ = intelligence quotient; SA = schizoaffective disorder; SCZ + S = schizophrenia with lifetime suicidal attempt; SCZ − S = schizophrenia without violent behavior or suicidal attempt; SE = standard error of the mean; ^*∗*^ SCZ + S > Healthy and SCZ − S (*p* < 0.05). ^*∗∗*^ SCZ − S < Healthy (*p* < 0.05); ^*∗∗∗*^ SCZ − S and SCZ + S > Healthy (*p* < 0.05).

**Table 2:** Activations in healthy subjects during the parametrically modulated Inflation and Success events.

Brain regions	L/R	BA	Talairach coordinates			Voxels (mm^3^)	Max *t*^*∗*^
			*x*	*y*	*z*		
[Inflation]							
Precentral gyrus/insula	R	44	42	17	7	1219	4.2
Insula	L	13	−30	20	7	345	3.9
Superior occipital gyrus	R	19	33	−73	28	697	−5.1
Cerebellum	R	-	15	−67	−29	498	−5.7
Precuneus	L	7	−3	−61	40	662	−5.4
Hippocampus	L	-	−24	−13	−11	998	−7.0
Cuneus	L	7	−21	−79	31	1667	−5.6
Precentral gyrus	L	4	−27	−25	46	563	−5.3
Superior temporal gyrus	L	21	−51	−4	−11	763	−5.9
[Success]							
Superior parietal gyrus, encompassing the left superior parietal gyrus, the bilateral occipital and cerebellum	R	7	30	−58	40	162420	13.3
Middle frontal gyrus, encompassing the right medial frontal gyrus	R	46	42	32	22	27327	10.3
Claustrum/putamen/insula	R	-	30	14	1	7534	8.0
Anterior cingulate gyrus	R	32	3	26	34	16158	10.9
Posterior cingulate gyrus	R	31	6	−31	31	1368	8.4
Parahippocampal gyrus	L	35	−15	−28	−5	1962	7.5
Claustrum/insula	L	-	−33	11	4	5209	7.2
Precentral gyrus	L	6	−30	−13	52	3045	6.5
Precentral gyrus	L	6	−51	2	34	4780	9.7
Middle frontal gyrus	L	9	−48	23	31	812	6.7

L/R = left/right hemisphere; BA = Brodmann area; SCZ = schizophrenia; ^*∗*^voxel-wise *p* < 0.001.

**Table 3:** Differences in activations between schizophrenia patients (with and without suicidal behavior) and healthy subjects during the parametrically modulated Inflation event.

Brain regions	L/R	BA	Talairach coordinates			Voxels (mm^3^)	Max *t*^*∗*^
			*x*	*y*	*z*		
[SCZ + S > SCZ − S]							
Cerebellar declive	R	-	18	−64	−20	920	4.85
Lingual gyrus	R	17	18	−85	1	458	4.48
[SCZ − S > SCZ + S]							
-							
[SCZ + S > Healthy]							
Superior temporal gyrus	L	38	−51	−1	−8	802	5.46
[Healthy > SCZ + S]							
-							
[SCZ − S > Healthy]							
-							
[Healthy > SCZ − S]							
-							

L/R = left/right hemisphere; BA = Brodmann area; SCZ = schizophrenia; ^*∗*^voxel-wise *p* < 0.001.

**Table 4:** Differences in activations between schizophrenia patients (with and without suicidal behavior) and healthy subjects during the parametrically modulated Success event.

Brain regions	L/R	BA	Talairach coordinates			Voxels (mm^3^)	Max *t*^*∗*^
			*x*	*y*	*z*		
[SCZ + S > SCZ − S]							
-							
[SCZ − S > SCZ + S]							
Medial frontal gyrus/anterior cingulate gyrus	R	10/32	18	44	7	363	4.99
[SCZ + S > Healthy]							
-							
[Healthy > SCZ + S]							
Culmen	R	-	21	−64	−23	1256	4.88
Cuneus	R	7	18	−76	31	441	4.86
Medial frontal gyrus/anterior cingulate gyrus	R	10/32	18	47	10	1034	5.27
Middle occipital gyrus	L	18	−30	−88	−2	575	4.38
[SCZ − S > Healthy]							
-							
[Healthy > SCZ − S]							
-							

L/R = left/right hemisphere; BA = Brodmann area; SCZ = schizophrenia; ^*∗*^voxel-wise *p* < 0.001.
